# Potentiation Physiologically Proven

**Published:** 1892-09

**Authors:** Gustav Jaeger

**Affiliations:** Stuttgart, Germany


### POTENTIATION PHYSIOLOGICALLY PROVEN.

BY PROF. DR. GUSTAV JAEGER, STUTTGART, GERMANY.

[Translated from the Leipzig *Allgem. Homœop. Zeitung*, Vol. 124, No. 11, by B. Fincke, M. D.]

Continued from page 348.

### IV. COMPARATIVE NEURAL ANALYSIS OF 17 ALKALI-SALTS.

*(a.) Preliminary Remark.*

Here is to be premised: *(a.)* the now following measurements have been made before the measurement described in Part I, as it were, for the sake of a first orientation. After finishing the measurements of Aurum-metallicum, Thuja, Aconitum, and Natrum-muriaticum which were published in my *Neural Analyse der homoeopathischen Verdunnungen*, Leipzig, 1881, and are now contained in the 3d edition of *Entdeckung der Seele*, there was not the least doubt with me that potentiation produces in all medicines, yea in all substances generally, the same changes to be produced physiologically, but in view of the great variety of substances it could possibly recommend itself to measure a larger number of them in order to see what variations thereby might ensue. In order to get clearness about this matter, I concluded to commence experiments with one group.

(*b.*) My choice fell upon the Alkali-salts for the following reasons :

*First*, because from them less properly *chemical* action of such kind as acids and alkalis or otherwise easily combining and decomposing substances produce may be expected. For the object of potentiation is not the *chemical* action as being in *direct* ratio to mass but the " *nervous* " action as being in *inverse* ratio to mass.

*Second*, since I wanted also to measure the *lower* potencies, of which at any rate *poisonous action* was to be expected, I chose a group of relatively *harmless* substances.

*Third*, I wanted to examine a group the chemical composition of which is well known and the physiological action of which is better known, because they are used frequently.

*Fourth*, I hoped to kill two flies with one stroke if I took a salt, because then the difference of the different *acids* as well as also that of the different *bases* would appear, so that it was not necessary to measure each separately. The following shall teach that this succeeded in a certain degree.

(*c.*) For these measurements I have this time *not swallowed* the medicine as was done with the potencies described in Part I of Kali-carbonicum, but I have only *inhaled* them. This was done for several reasons ; in order to be able to compare several substances, which was the main object, it is necessary to measure them under most possibly equal relations, and this requires that they be measured as quickly as possible in succession on one and the same day ; but this can generally be done only when the first substance is removed from the body rapidly enough and as completely as possible. In this regard my long practice has given me sufficient experience that substances incorporated by inhalation disappear much more rapidly than such as had been swallowed, hence I preferred the mode of inhalation. Thus it was possible to institute safe measurements of lower potencies, of up to seven substances, within two hours a day. Higher degrees of potentiation allow even a more rapid procedure. Finally, the economy of time had to be considered ; in order to obtain a general view more than 100 substances must be measured, and in order to try all of them, as I did with Kali-carbonicum, a time is needed over which I cannot dispose; besides it is in the interest of the reader to receive this view as soon as possible, for: *bis dat qui cito dat.*

(*d.*) Likewise in the interest of the time of reader and author I have limited myself to measure for every potency of each substance a single decade-mean (composed of 40 acts) and from it to form only *one number*, not a *whole series* of *numbers*, as in the measurement of Kali-carbonicum. The numbers of the following tables, therefore, arose in this manner: a rest-number (these are stated in Table I and II) formed from four decades; then I measured immediately a *medicine-number* in the same manner. To measure each time first an *alcohol-number*, similarly as I measured each time a water-number in Kali-carb., I omitted on account of saving time, and then because the alcohol-number differed very little from my rest-number (the difference between rest and alcohol-number amounted in seven experiments *seriatim* +25%, +2%, +1.6%, +1.4%, +2.1%, +1%, +1.7%). Then it was superfluous, because every potency and every substance contained the same alcohol, so that the result of comparison was not changed by it. The tables again contain not the medicine-number itself, but *the difference in per cent. between it and the rest-number* which of course again is either a *plus* or *minus* value.

(*b.*) *Source of Error.*

(1.) The limits of error are not always the same, but they vary with the nervous disposition; therefore in measurements of great exactness, where small differences are to be ascertained, the concerning quantity must be determined each time. This, to be sure, is not the case in our subject, for the differences obtained from the medicines are so extraordinarily great that even in the most unfavorable disposition they are to be found. This first reason, therefore, is not necessary to dwell upon any further, but the following circumstance is to be added:

(2.) That no man ever succeeds in overcoming the prejudice of *learned* circles against an innovation is the experience of all the centuries, but that it is not impossible to penetrate the circles of the *practical* men, my experiences in the field of habiliment have taught. Now, a practice of ten years with neural analysis, which I executed, not only upon scientific ground, but directly in my intercourse with the practical men, and for practical purposes, has instructed me that this method is exceedingly valuable for practice, and then, in that the practical men would soon learn to estimate and *fear* its superiority. The object in my practice was (as I showed in No. I) to watch the manufacturing industry allied with me, in order to protect it quickly and safely against intentional or unintentional contaminations of the manufactured goods. In this way I convinced myself and my people of the almost uncanny safety and exactness, *e. g.*, a thread of a few centimeters sufficed to determine in a few seconds whether it was treated with a noxious color, or contaminated in other ways. It is, however, remarkable that the method, in spite of my frequent publications, has been completely ignored by those practical men who should have the most frequent opportunity and occasion to use it, viz., the chemists who are in the service of the Boards of Health. In the expectation that these lines may fall in the hands of one or the other of these gentlemen, I will here give a more exact explanation of the source of error, for only, if this is contrasted with the numbers obtained from the measured objects, an idea of the exactness of the method can be obtained. Finally, this is not only valid *extra muros*. I hope later to be enabled to show what eminently practical application the neural analysis can obtain as a reagent of the degree of potentiation of homœopathic remedies.

[TO BE CONTINUED.]

